# Global Challenges in the Provision of Theranostic Services the International Atomic Energy Agency (IAEA) Perspective

**DOI:** 10.1055/s-0045-1807251

**Published:** 2025-04-17

**Authors:** Anita Brink, Juliano J. Cerci, Francesco Giammarile, Danylo Yurystovskyi, Aruna Korde, Enrique Estrada-Lobato, Miriam Mikhail-Lette, Olivier Pellet, Homer A. Macapinlac, Diana Paez

**Affiliations:** 1 International Atomic Energy Agency, Nuclear Applications Human Health, Wien, Vienna, Austria; 2 Quanta Diagnostico e Terapia, Curitiba, Brazil

**Keywords:** global challenges, IMAGINE database, International Atomic Energy Agency, NUMDAB database, theranostics

## Abstract

The global expansion of theranostics, a field combining molecular imaging and targeted therapy, has accelerated with the Food and Drug Administration approvals of Lutathera and Pluvicto. However, significant challenges hinder equitable access, particularly in low- and middle-income countries (LMICs). These include infrastructure limitations, such as the high cost of gamma cameras, positron emission tomography/computed tomography scanners, cyclotrons, and maintenance, as well as disparities in the availability of radiopharmaceuticals. In LMICs, one single-photon emission computerized tomography scanner may serve 33 million people, compared with 57,000 in high-income countries. Furthermore, shortages of key isotopes like 99Mo and 177Lu highlight vulnerabilities in global supply chains.

Staffing and training are critical issues, as delivering theranostic services requires specialized teams that are often scarce in LMICs. The International Atomic Energy Agency (IAEA) addresses these challenges by offering training programs, developing databases like IMAGINE and NUMDAB, and fostering technical cooperation among member states. To improve access to theranostic services, strategies such as using alternative imaging methods and enhancing local radiopharmaceutical production are recommended.

The IAEA's Rays of Hope initiative aims to strengthen regional capacities, improve infrastructure, and address the critical shortage of trained professionals. A coordinated global effort is essential to overcome these barriers, reduce costs, and ensure equitable access to theranostics, particularly in resource-limited settings.

## Introduction

The concept of theranostics is not new. In 1941, Saul Hertz successfully treated the first metastatic thyroid cancer patient with ^131^ iodine. 1 In the last few years, the field of theranostics has expanded significantly with the advent of ^177^ Lu-DOTATATE and ^177^ Lu-prostate-specific membrane antigen (PSMA) therapies. The definition of theranostics has evolved to “a combination of molecular targeted imaging and therapy in which imaging provides actionable information that enables new or more effective therapies.” 2

The Food and Drug Administration approval of Lutathera (177 Lu-DOTATATE) in 2018 and Pluvicto ( ^177^ Lu vipivotide tetraxetan, ^177^ Lu-PSMA-617) in 2023 has accelerated the growth of theranostics worldwide. 3 4 5 This article will review the challenges that need to be addressed to ensure safe and sustainable theranostic service delivery globally. It aims to underscore the challenges in implementing a theranostics program and it will contrast the difficulties faced by low- and middle-income countries (LMICs). It will also point the reader to useful guidance documents and tools on theranostics developed by the International Atomic Energy Agency (IAEA).

## A Global Perspective on the Current Status of Theranostics

To understand the needs and challenges in the field, the IAEA continuously assess the status of nuclear medicine (NM) and diagnostic imaging globally. This ongoing evaluation uniquely positions the IAEA to identify the obstacles faced by current providers of theranostic services and barriers that new service providers must overcome to establish such a service.

One of the mechanisms the IAEA has put in place to monitor the status of NM and diagnostic imaging is a series of databases. The IAEA Medical imaging and Nuclear mEdicine global resource database (IMAGINE), launched in 2021 and accessible through Human Health Campus is a comprehensive compilation of estimated medical imaging resources for both radiology and NM, containing information on infrastructure from over 170 countries. 6 A separate database NUMDAB (NUclear Medicine DAtaBase), launched in 2006 and accessible through NUMDAB – Human Health gathers information about the status of NM service delivery at a facility-based practice level. 7 Both databases are constantly updated from multiple sources including: questionnaires completed or presentations delivered by country representatives who participate in national, regional, and interregional IAEA technical cooperation projects; IAEA technical officers as through country mission reports; other agencies in the United Nations system; authorities/official bodies; scientific publications; international conferences including those hosted by the IAEA; professional societies; industry trade association reports on the install base; and market reports. 7

According to IMAGINE, more than 140 countries have single-photon emission computerized tomography (SPECT) or SPECT/computed tomography (CT) availability, with approximately 27,000 systems installed, while 109 have positron emission tomography (PET)/CT and more than 5,200 PET/CT systems are installed. The data shows a clear disparity in theranostic service delivery between high-income countries and LMICs. While one SPECT scanner serves approximately 57,000 people in high-income-countries, one scanner serves an average of 33 million people in low-income countries. The differences become even more striking when we consider that a PET scanner serves an average of 250,000 people in high-income countries, while one scanner serves a staggering 250 million people in low-income countries. 6

According to the IMAGINE database, 89 countries have cyclotrons primarily to produce ^18^ fluorine. Additionally, the IAEA maintains a comprehensive database on the availability of cyclotrons through the IAEA Accelerator Knowledge Portal section on “Cyclotrons used for Radionuclide Production” accessible through Pages - Cyclotrons used for Radionuclide Production. As of 2023, there were over 1,500 cyclotrons installed worldwide. Of these approximately 1,400 have energies of less than 25 MeV and approximately 130 have energies between 25 and 70 MeV. 8

Another IAEA initiative, which assessed the availability of radiopharmaceuticals globally, was a survey conducted by the IAEA, which began in 2023 and concluded in April 2024. This survey addressed various aspects, including the availability of devices, specialized personnel, access to radioisotopes and radiopharmaceuticals, domestic production capacities, as well as the availability of radiopharmaceuticals and radiopharmaceuticals for therapeutics. Challenges related to access to radiopharmaceuticals were also explored. In addition, the survey focused on regulatory issues and the specific challenges faced by participants. The survey results were recently published (November 2024) in the *Lancet Oncology* commission on Radiotherapy and Theranostics. 9

A total of 105 countries participated in this survey. Among the participating countries, the most common therapy was ^131^ iodine for well-differentiated thyroid cancer, with approximately 250,000 therapies performed annually in 80 countries. Therapies for the palliation of bone pain were performed in approximately 60 countries; the most widely used radiopharmaceuticals were ^153^ Sm, followed by ^89^ Sr, ^223^ Ra, and ^32^ P, with a total of close to 45,000 therapies per year.

In terms of therapies for neuroendocrine tumors (NETs), 39 countries performed around 43,000 targeted radionuclide therapies with ^177^ Lu- DOTATATE and approximately 30 countries perform ^131^ I meta-iodobenzylguanidine therapies, with approximately 1,900 procedures per year.

Targeted radionuclide therapies with ^177^ Lu PSMA for metastatic prostate cancer are being performed in 32 countries with close to 48,000 therapies per year. In addition, 12 countries performed close to a 1,000 ^225^ Ac PSMA therapies per year.

Only 10 reported performing liver therapies with microspheres, with a total of 24,000 procedures per year.

## Factors Limiting Access to Nuclear Medicine Diagnostic Services

### Infrastructure

LMICs face significant infrastructure challenges for establishing theranostic services. The capital cost of installing a gamma camera, PET/CT scanner, and cyclotron can be prohibitive. Once the equipment is installed, routine maintenance is essential. Unfortunately, many countries struggle with the cost associated with maintenance contracts. In nations with a limited number of installed units, service engineers are often not based locally. Consequently, if maintenance or repairs are needed, engineers must travel significant distances, and the availability and cost of spare parts, as well as shipping times, could significantly impact on service delivery. 10

Due to the relatively short half-lives of the radiopharmaceuticals used in theranostics, adequate infrastructure is essential to deliver the radiopharmaceuticals. In many countries supplies of radiopharmaceutical come only from a single provider, which increases the susceptibility to supply chain disruptions. 11

The use of generators could mitigate some of the logistical challenges. The ^99^ Mo/ ^99m^ Tc generator is the mainstay of everyday NM practice, particularly because ^99m^ Tc is used in over 90% of studies performed with gamma cameras, which are more widely available than PET scanners. 6 The ^68^ Ge/ ^68^ Ga generator enables in-house labeling of ^68^ Ga-PSMA and ^68^ Ga DOTATATE for PET/CT imaging. Several therapeutic radionuclides can also be generator-produced such as ^90^ Y, ^166^ Ho, ^188^ Re, ^211^ At, ^212^ Pb, ^213^ Bi, and ^225^ Ac. One of the limitations of generators is the number of doses that can be prepared, usually about of 3 to 6 doses per day. 11

Cyclotron-produced radiopharmaceuticals typically have relatively short half-lives, which limits the distribution to remote practices. Most cyclotrons installed globally are lower energy cyclotrons with the focus of producing ^11^ C and ^18^ F. Careful infrastructure planning ensures that ^18^ F products can be delivered to other PET/CT facilities in a region. 11

In addition to considering aspects related to production, it is important to consider those related to transportation, the management of radioactive waste, and all regulations concerning radiation protection for patients, staff, the general public, and the environment. In these fields, the IAEA also provides a series of guides and documents to support member states in proper management. 12

### Strategies Increase Availability of Pretreatment Imaging Assessment

It is well understood that tumor biology not only differs between patients but significant heterogeneity in tumor biology is frequently seen within individual patients. This can manifest as metastatic lesions, which express different tumor characteristics and even with heterogeneity of receptor expression seen in a single mass lesion. 13 Pretreatment imaging is essential to identify the patients who will benefit the most from these treatments. This is particularly crucial in the resource-constrained settings, where resources should be directed to those patients for whom the treatment would have the greatest impact.

In most high-income countries imaging for NETs is done with ^68^ Ga DOTATATE and ^18^ F fluorodeoxyglucose PET/CT. However, in LMIC many practices utilize ^99m^ Tc EDDA/HYNIC TOC (Tektrotyd), a SPECT radiopharmaceutical, as an alternative to assess whether a tumor has adequate somatostatin receptor expression for treatment. 14 15 Additionally, ^99m^ Tc-labeled HYNIC-IPSMA allows for SPECT imaging in prostate and other cancers providing a viable solution when PET/CT services with ^18^ F PSMA or ^68^ Ga PSMA are unavailable. 16 17

## Factors Limiting Access to Targeted Radionuclide Therapies

### Staffing Needs

The field of NM is highly specialized, and the staffing needs are dictated by the department's service delivery. As departments expand their services from providing diagnostic imaging alone to therapies the staffing demands increase accordingly. A department that delivers theranostic services requires a diverse team, including nuclear physicians, NM technologists, at least one medical physicist, specialized nursing staff, and administrative staff. Additionally, radiopharmacists are recommended for departments that compound radiopharmaceutical products, particularly those operating a level 3 radiopharmacy. 18

To help departments calculate their staffing needs, the IAEA has developed the staffing needs calculator, which is available online. 19 20 A separate online tool is available to assess the staffing requirements of medical physics personnel, including the staff specific to NM departments. 21

One of the significant challenges in delivering effective theranostic services is the demand for highly specialized skills. This results in elevated staffing cost and a shortage of qualified professionals in many departments. In LMICs, these challenges are further compounded by limited remuneration and career opportunities for highly trained staff. Consequently, there is a tendency for skilled professionals to migrate to regions offering better compensation and a chance to engage in cutting-edge medicine and research, contributing to the “brain drain” phenomenon. 10

### Training Needs and Opportunities

The training requirements of the team delivering the theranostic service are evolving alongside the expansion of service delivery. In response to this, the IAEA has developed a guidance document that outlines the education and practice standards for theranostics. This document specifies the minimum training and the numbers of theranostic procedures required for credentialing. 22

The IAEA facilitates training of personnel from member states through short- (between 2 weeks and 6 months) and long-term fellowships or residencies (up to 4 years). Member states identify their specific needs, which can range from establishing a basic theranostic service, often limited to ^131^ I therapy for benign and malignant thyroid disease, to a comprehensive service, which includes both peptide receptor ligand therapy and peptide receptor radionuclide therapy. The IAEA works with the member state to develop a proposal aimed at strengthening the desired service. Suitable candidates for training are identified by the member state and the IAEA assist by placing these candidates in centers that meet the required theranostic training capability as set out in the white paper on the guiding principles on the education and practice of theranostics. 22

While IAEA training programs provide excellent opportunities, fellows particularly those in long-term fellowships face significant challenges. These fellowships take place in countries outside their home nation, requiring participants, many of whom are young professionals with families, to leave behind spouses and young children. Language barriers, cultural differences, and the uncertainty of employment upon returning to the home countries, despite prior promises, add to the difficulties faced by the fellows. 23

There are a limited number of training facilities in LMICs, reflecting the broader shortage of radiation medicine infrastructure, including NM, in these regions. The training capacity of these centers is also closely tied to the availability of skilled professionals, which is often constrained by staffing costs in many countries.

The IAEA has recently launched the Rays of Hope (RoH) initiative. This initiative is aimed at assisting member states in expanding their capacity in radiotherapy and multimodality imaging. Central to this initiative are the regional RoH Anchor Centres. The RoH Anchor Centres serve as training and knowledge hubs, which in turn strengthen education and training in the region. 24

Additionally, female students interested in pursuing a career in NM at master's level are also eligible for the Marie Sklodowska-Curie Fellowship Program (MSCFP). This program offers scholarships for master's studies and provides opportunities for internships facilitated by the IAEA, empowering motivated female students to advance in the field. 25

### Availability of Radiopharmaceuticals

In the last two decades the NM community has frequently faced supply chain disruptions in the production and delivery of ^99^ Mo/ ^99m^ Tc generators. Shortages occurred in 2009 to 2010, 2017, 2018, 2022, and more recently the last quarter of 2024. 26 27 28 29 These shortages were caused due to unexpected shutdown of reactors and extended periods of refurbishment. Many of the facilities producing ^99^ Mo are old and a number have closed since 2009. 30 By the end of 2023, NorthStar Medical Radioisotopes, the only commercial producer of ^99^ Mo in the United States, shut down its ^99^ Mo production facility, citing increasing cost and competition. 31

The breakdown in research reactors also affected the supply of ^131^ I and in 2022 there was a global shortage of ^131^ I capsules, which lasted for more than a year. 32

There are also concerns that the availability of ^177^ Lu will not be sufficient in the long term. 33 In 2023 there was a shortage of ^177^ Lu-PSMA-617 (Pluvicto) that lasted nearly 7 months. To meet the demand to this product Novartis had to double the production. Novartis has an annual capacity target of at least 250,000 doses per year. Currently, the treatment protocols comprise of four or five doses given 6 weeks apart. 34 Thus, Novartis produces only enough to treat around 50,000 to 62,500 patients annually. In contrast, it is estimated that globally 158,162 patients were eligible for ^177^ Lu PSMA therapy in 2024. 9 Globally, an increase in ^177^ Lu production will be needed to meet the increasing demand. 35

### Cost of Targeted Radionuclide Therapies

The recent *Lancet Oncology* commission on Radiotherapy and Theranostics showed that for nine countries (Australia, India, Netherlands, Germany, the United States, South Africa, Thailand, Jordan, and the Philippines), the projected social cost-saving impact of adding ^177^ Lu PSMA to the standard of care would be $725 million over 7 years. 9

However, the current cost of the therapies places it out of reach for many patients especially in LMICs. A Dutch study estimated that the per patient cost in their country equated to €47,546 if using the Vision regimen and €35,886 if using the SPLASH regimen. 36 In the United States, it is estimated that treatment using ^177^ Lu PSMA led to an increased in cost compared with standard of care, $169,110 versus $85,398. The corresponding cost effectiveness ratio for this therapy was $200,000 per quality-adjusted life years. 37 It should be noted that there is a large variation in the cost of radiopharmaceuticals; for instance, in India 100 mCi ^177^ Lu-PSMA-617 costs 80,000 INR (±€900) contrasted with $42,500 per cycle of Pluvicto in the United States. 3 35

In many countries this treatment is not available in the national health system, which means that the patient must fund the treatment privately or through medical insurance.

Health technology assessments are essential to ensure that targeted radionuclide treatments are included as treatment options in national health systems. These assessments also play a crucial role in motivating medical insurers to fund these treatments. 38

### Regulatory Issues

The main challenge associated with the introduction of new radiopharmaceuticals is that both radiopharmaceuticals used for diagnostic and therapeutic purposes are considered medicinal products and are therefore subject to regulation by health authorities, just like other medicinal products, to ensure that they are safe, effective, and produced under quality-controlled conditions. 39

Unfortunately, in many countries, there is a lack of clarity and guidelines regarding the rules and regulations governing the approval and registration of radiolabeled medicines. Alternatively, the demands are so high that they make it impossible to access new diagnostic or therapeutic procedures, leaving their use under compassionate care use.

To support member states the IAEA published the Good Practice for Introducing Radiopharmaceuticals for Clinical Use in 2016, endorsed by several professional organizations, the publication seeks to provide support to countries and institutions that are introducing new radiopharmaceuticals. The aim of this publication is to review practices in different countries, explain the necessary steps, and provide references for human studies with new radiopharmaceuticals whose quality, safety, and efficacy have already been established in other countries. 40

In this fast-evolving field of theranostics the safety of the public, staff, and patients is of paramount importance. 41

Several organizations have produced guidance documents on the practice of theranostics. In 2022, the European Association of Nuclear Medicine, Society of Nuclear Medicine and Molecular Imaging, and IAEA produced a joint guide on how to set up a theranostic center. 42

Several guidelines published by international societies are available to guide a variety of treatments such as ^177^ Lu PSMA, ^177^ Lu DOTATATE, and others. 43 44 45

## Conclusion

To ensure equitable access to theranostic services a multifaceted approach is required, which addresses infrastructure, staff, and training needs. Efforts must be made to improve the availability and reduce the cost of targeted radionuclide therapies. All of these actions should occur within a well-regulated framework.

Through a comprehensive and multidisciplinary approach, the IAEA plays a fundamental role in supporting member states to address all challenges to achieving equitable and quality access to targeted radionuclide therapies, which includes:

- Radiopharmaceutical production: Ensuring the availability and quality of radiopharmaceuticals.
- Transportation and waste management: Addressing the logistical challenges and ensuring safe disposal.
- Radiological protection: Implementing measures to protect patients, staff, and the environment.
- Clinical applications: Promoting the effective use of radiopharmaceuticals in clinical settings.
- Education and training: Supporting the training and education of professionals in this field.
- Continuous medical education: Providing ongoing education and training for health care professionals.
- International cooperation: Fostering collaboration between countries to share knowledge and resources.
- Improving access: Working to make radiopharmaceutical therapies more accessible and affordable.

By leveraging the immense potential within the global NM community, we can achieve worldwide theranostic service delivery.
